# Condensing Effect of Cholesterol on hBest1/POPC and hBest1/SM Langmuir Monolayers

**DOI:** 10.3390/membranes11010052

**Published:** 2021-01-13

**Authors:** Pavel Videv, Nikola Mladenov, Tonya Andreeva, Kirilka Mladenova, Veselina Moskova-Doumanova, Georgi Nikolaev, Svetla D. Petrova, Jordan A. Doumanov

**Affiliations:** 1 Faculty of Biology, Sofia University “St. Kliment Ohridski”, 8 Dragan Tzankov Blvd., 1164 Sofia, Bulgaria; pvidev@biofac.uni-sofia.bg (P.V.); nikola.mladenov@abv.bg (N.M.); k_mladenova@biofac.uni-sofia.bg (K.M.); moskova@biofac.uni-sofia.bg (V.M.-D.); gn_georgiev@uni-sofia.bg (G.N.); spetrova@biofac.uni-sofia.bg (S.D.P.); 2 Faculty of Medicine, Medical University-Sofia, 1 Sv. Georgi Sofiiski Str., 1431 Sofia, Bulgaria; 3 Institute of Biophysics and Biomedical Engineering, Bulgarian Academy of Sciences, Acad. G. Bonchev Str., Bl. 21, 1113 Sofia, Bulgaria; t_andreeva@abv.bg; 4 Faculty of Applied Chemistry, Reutlingen University, Alteburgstraße 150, 72762 Reutlingen, Germany

**Keywords:** hBest1, cholesterol, sphingomyelin, POPC, Langmuir monolayers, condensing effect

## Abstract

Human bestrophin-1 protein (hBest1) is a transmembrane channel associated with the calcium-dependent transport of chloride ions in the retinal pigment epithelium as well as with the transport of glutamate and GABA in nerve cells. Interactions between hBest1, sphingomyelins, phosphatidylcholines and cholesterol are crucial for hBest1 association with cell membrane domains and its biological functions. As cholesterol plays a key role in the formation of lipid rafts, motional ordering of lipids and modeling/remodeling of the lateral membrane structure, we examined the effect of different cholesterol concentrations on the surface tension of hBest1/POPC (1-palmitoyl-2-oleoyl-sn-glycero-3-phosphocholine) and hBest1/SM Langmuir monolayers in the presence/absence of Ca^2+^ ions using surface pressure measurements and Brewster angle microscopy studies. Here, we report that cholesterol: (1) has negligible condensing effect on pure hBest1 monolayers detected mainly in the presence of Ca^2+^ ions, and; (2) induces a condensing effect on composite hBest1/POPC and hBest1/SM monolayers. These results offer evidence for the significance of intermolecular protein–lipid interactions for the conformational dynamics of hBest1 and its biological functions as multimeric ion channel.

## 1. Introduction

Bestrophin-1 (hBest1) is a transmembrane protein that is expressed on the basolateral membrane of cells of the retinal pigment epithelium (RPE) and neuronal cells. One of the main functions of hBest1 is the calcium-dependent transport of chloride ions [1]. It is also thought to play a role as a channel in the central nervous system, where it is involved in the transport of γ-aminobutyrate (GABA) in glial cells and glutamate (Glu) in astrocytes and neurons [2,3].

Mutations in the *BEST1* gene (manifested in the synthesized protein) are responsible for the development of retinal diseases referred as Bestrophinopathies [4,5,6,7,8,9,10,11,12,13].

Scientific data about the molecular interactions of hBest1 with different cell membrane lipids are insufficient in elucidating the functional activity of the protein and especially with cholesterol (Chol), which plays a key role in the formation of “classic” lipid rafts [14,15,16,17,18]. On the other side, the interactions of sphingomyelins (SM) and phosphatidylcholines (PC) (make up more than 50% of membrane lipids) with cholesterol are essential for the appropriate lateral structure of biological membranes and conformational protein dynamics [19,20]. Experiments with liposomes and bilayers using membrane lipids showed that regardless of the conditions, Chol display a well-defined affinity for SM (e.g., Chol forms dimers with SM) and ability to increase lipid order [21,22]. Cholesterol can be localized equally well in both ordered and disordered membrane regions, often associated with its role in some types of membrane sorting [23,24].

Cholesterol’s ability to “thicken” the hydrophobic region of the membrane is a prerequisite for creating favorable conditions for localization and accumulation of proteins in this region (hydrophobic sorting) [25].

One of the main physicochemical properties of cholesterol is its condensing effect. The interactions of Chol with lecithin induces conformational changes and decrease of “disturbances” in the hydrocarbon residues of fatty acids, their orientation turns towards the central axis of the molecule and a reduction in free volume and cross section occurs, as well as reduction of the area occupied by the molecules without changing the surface pressure [26,27]. Cholesterol has no condensing effect when interacting with some types of polyunsaturated phospholipids [27].

The condensing effect of Chol affects the functions of membrane proteins, such as Na^+^ K^+^ ATPase, rhodopsin, Ca^2+^ ATPase, etc. [28,29]. 

In this study, a special attention is paid to Chol interactions with hBest1 in pure and composite protein–lipid model systems.

Using Langmuir monolayers for surface pressure measurements and Brewster angle microscopy imaging, we prove that Chol displays negligible condensing effect on pure hBest1 monolayers which becomes higher in the presence of Ca^2+^ ions. Chol displays also a significant condensing effect on composite hBest1/POPC and hBest1/SM monolayers in the absence of Ca^2+^, which differs after the addition of Ca^2+^ ions. 

Our results shed light on the Chol role for the organization and structural conformation of hBest1 molecules in the plane of surface monolayers in order to understand protein association with cell membrane domains, a process that probably influences its biological functions. 

## 2. Materials and Methods 

All reagents and chemicals were purchased from Sigma-Aldrich (St. Louis, MO, USA) unless otherwise indicated.

### 2.1. Cell Cultures and hbest1 Purification

The hBest1 protein was produced in MDCK II (ATCC, CRL-2936) cells stably transfected with hBest1 (MDCK-hBest1) [30,31]. The processes of cell culturing, hBest1 extraction and purification are performed as described in [31]. The concentration of the purified hBest1 was determined using the method of Smith et al. [32].

### 2.2. Monolayers Experiments

The experiments were executed at identical experimental conditions using a Langmuir balance (Kibron Inc., Helsinki, Finland), equipped with PTFE coated stainless steel multiwell plate (well capacity 0.5 mL) and Wilhelmy dynamometric probe, at 35 ± 2 °C. The monolayers were formed on a subphase containing 150 mM NaCl (pH ~7) with and without addition of 0.5 μM CaCl_2_ [33]. The components of the monolayers were applied consecutively to the interface: first, POPC or egg SM (1 mM stock solutions in chloroform) were spread until surface pressure π reaches 20 mN/m, and Chol (0.1 mM stock solution in chloroform) was added 2 min later with molar fraction X_Chol_ 0.167 0.285, 0.375, 0.444 or 0.5. After a further 2 min, when the solvent had evaporated and π reached a constant value, hBest1 (1 mg/mL stock solution in 150 mM NaCl) was added to the binary POPC/Chol or SM/Chol monolayers. The volume of the spread hBest1 was calculated with respect to the main lipid in the system (POPC or SM) with molar ratio 1:45 and 1:86, respectively, so that the area occupied by the protein to that occupied by the surrounding lipids is 1:3 [34]. The measurements were carried out 10 min after the hBest1 has been applied on the interface. Each experiment was performed at least seven times. Values are expressed as a mean ± SE. The *p*-value was calculated using Student’s *t*-test. The calculated *p* values based on our data are less than 0.005. Data with *p* < 0.05 are considered statistically significant. 

### 2.3. Brewster Angle Microscopy Studies

The monolayers were visualized by BAM (UltraBAM, Accurion GmbH, Göttingen, Germany), which combines real-time imaging and high resolution (lateral resolution down to 2 μm). The experiments were conducted at 35 ± 2 °C; p-polarized red light from a 50 mW broadband laser source was directed at the Brewster angle to the aqueous surface giving zero reflectivity. The monolayers were formed as described in Section 2.2. The images were taken 10 min after hBest1 has been applied. 

## 3. Results

### 3.1. Condensing Effect of Cholesterol on hbest1, hbest1/POPC and hbest1/SM Monolayers

**Figure 1:**
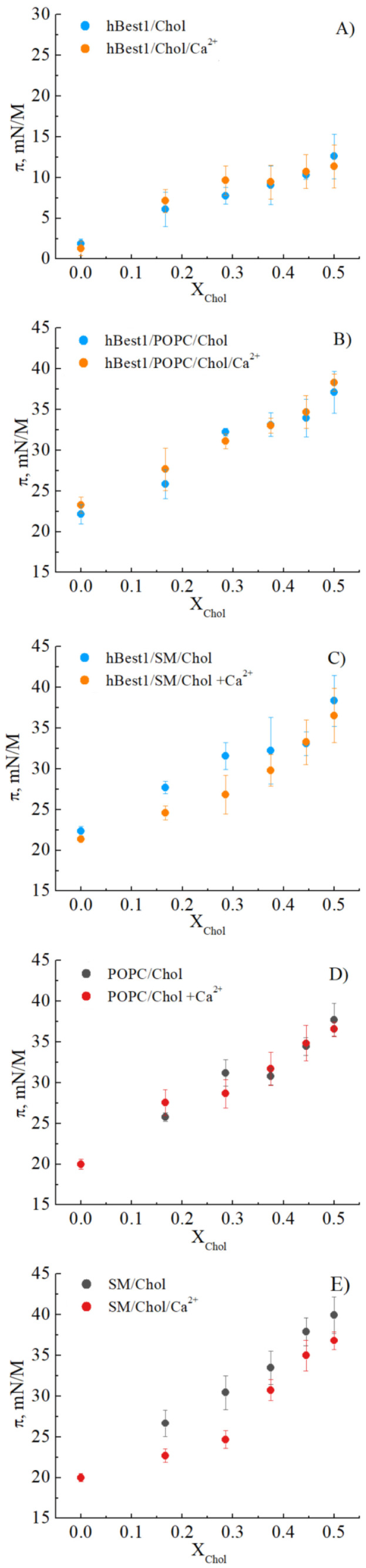
Condensing effect of cholesterol on (**A**) hBest1, (**B**) hBest1/POPC, (**C**) hBest1/SM, (**D**) POPC and (**E**) SM monolayers (at molar fractions of Chol 0.167; 0.285; 0.375; 0.444 and 0.5) in the presence or absence of Ca^2+^, at 35 ± 2 °C. Data are presented as mean ± SE, n = 7.

Cholesterol exerts a negligible condensing effect on pure hBest1 monolayers, however the addition of Ca^2+^ ions considerably enhances this effect (Figure 1A). The increase of X_Chol_ from 0.29 to 0.38 is not accompanied by an elevation of the surface pressure, as expected from the π/A isotherm of Chol—very steep, characterized by a sharp increase of π with a decrease of mean molecular area, described in [35]. With increasing the molar Chol fractions from 0.29 to 0.38, the surface pressure slightly decreases.

The condensing effect of Chol on POPC monolayers is already well studied and the results correspond well to our data (Figure 1D). A very strong condensing effect is found at X_Chol_ = 0.38, exactly at the same molar fraction of Chol reported in a previous thermodynamic study of this system [36]. Addition of Ca^2+^ ions supports the condensing activity of Chol and it appears earlier, at X_Chol_ = 0.29 (Figure 1D), but is less pronounced. The effect of Chol on the hBest1/POPC (1/45) monolayers resembles the one observed on the mono-component hBest1 and POPC monolayers and predominates at X_Chol_ = 0.38. Our previous study showed that the components in the binary hBest1/POPC (1/45) monolayers, both in presence and absence of Ca^2+^ ions [37] do not cause a condensing effect. Therefore, the condensation in the ternary hBest1/POPC/Chol monolayers results from the addition of Chol. Addition of Ca^2+^ ions does not affect the condensing potential of Chol in this ternary system and the two π/X_Chol_ curves in Figure 1B nearly match.

In the SM/Chol monolayers the condensing effect of Ca^2+^ ions enhance the one of Chol (Figure 1E). The effect emerges at X_Chol_ = 0.17, reaches a maximum at X_Chol_ = 0.29, then weakens with a further increase in the molar fraction of Chol and disappear at X_Chol_ > 0.44 (Figure 1E). The effect is cumulative and arises from the condensing effect of Ca^2+^ ions on SM monolayers [38,39] and on Chol monolayers [40]. The binary hBest1/SM (1/86) monolayers are nearly ideally mixed and do not evidence condensation at mixing, as proved by the total free energy of mixing in presence and absence of Ca^2+^ [41]. The most prominent is the condensing effect of Chol in the ternary hBest1/SM/ Chol monolayers. In the absence of Ca^2+^ ions it appears at 0.29 < X_Chol_ < 0.5. Addition of Ca^2+^ significantly intensifies the condensing effect and it manifests itself in the whole region of X_Chol_ between 0 and 0.5 (Figure 1C).

### 3.2. Morphology of hBest1/Chol, hBest1/POPC/Chol and hBest1/SM/Chol Monolayers

**Figure 2:**
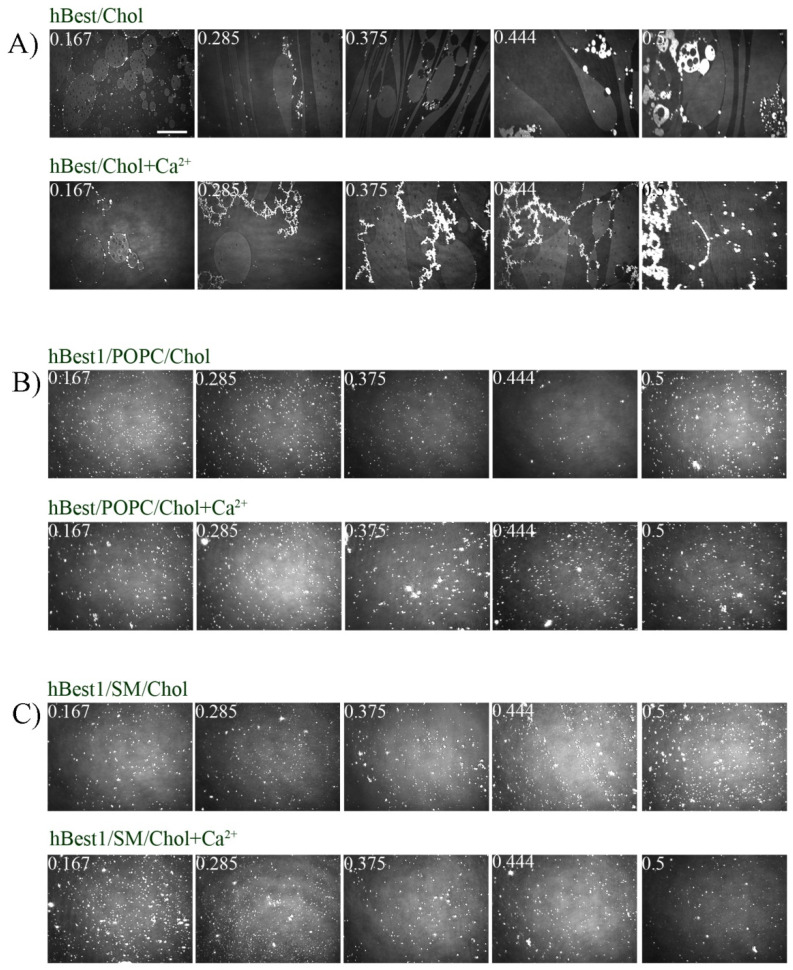
BAM images of (**A**) hBest1/Chol monolayers, (**B**) hBest1/POPC/Chol, and (**C**) hBest1/SM/Chol monolayers (at molar fractions of Chol 0.167; 0.285; 0.375; 0.444 and 0.5) in the presence or absence of Ca^2+^, at 35 ± 2 °C. The white scale bar = 100 µm.

The morphology of the equilibrium multicomponent monolayers was investigated by BAM and presented in Figure 2. All pictures in Figure 2A show the coexistence of gas (black areas) and condensed (gray areas) phase, which is typical for Chol but only at high molecular areas and π ≈ 0 mN/m. The equilibrium surface pressure of the binary hBest1/Chol monolayers ranges between 6 and 13 mN/m, where Chol monolayers were shown to present a homogeneous condensed phase [42]. hBest1 monolayers have been shown to be compact and homogeneous from spreading to the end of the compression at 20 mN/m [33]. However, an additional more condensed phase, characterized by very bright oval-shaped domains, is present in hBest1/Chol monolayers and the area occupied by this phase expands with increase of the molar fraction of Chol (Figure 2A). Ca^2+^ ions provoke a slight condensation of hBest1 monolayers but it is not accompanied by appearance of bright domains [33]. It is clear that Chol molecules induce a condensation of hBest1 monolayer (it is possible hBest1 to induce a condensation of Chol monolayer) and this effect is enhanced by the presence of Ca^2+^ ions. We have already demonstrated that Ca^2+^ has condensation effect on hBest1 monolayers and cause changes in the conformation and oligomerization of protein molecules [33]. One very recent study revealed that Chol molecules in Langmuir monolayers exist in two different solid phases differing by the orientation of the flexible isooctyl chain attached to C17—tilted or vertical [43]. We consider that hBest1 molecules induce a condensation of Chol, and Ca^2+^ ions boost this effect.

Addition of the chaotropic Ca^2+^ ions disrupts the hydration shell of the POPC zwitterionic polar head and contribute to the formation of small bright POPC domains with higher molecular density [33]. The addition of hBest1 with molar ratio hBest1/POPC = 1/45 provokes some condensation, leading to small bright domains appearance [33]. As both effects accumulate, in the presence of Ca^2+^, the binary hBest1/POPC monolayers show more numerous bright domains. Here, we demonstrate that the incorporation of Chol with different molar fractions into the binary hBest1/POPC (1/45) monolayers does not alter their morphology. The same numerous bright domains with a relatively uniform size are evenly distributed in the homogeneous condensed phase (Figure 2B). 

It has been shown that binary hBest1/SM monolayers (molecular ratio 1/86) are compact and homogeneous at π > 15 mN/m [41]. Images in Figure 2C demonstrate the condensing effect of Chol on hBest1/SM (1/86) monolayers expressed by the formation of numerous small bright domains of highly condensed phase equally distributed in the compact homogeneous monolayer. These results are fully in line with the data in Figure 1.

In addition, BAM images of POPC/Chol and SM/Chol monolayers, in the presence/absence of Ca^2+^, are shown as Supplementary Data (Figure S1).

## 4. Discussion

The concentration of cholesterol in membranes can affect many physicochemical, biochemical and biophysical properties of membrane proteins. For Na^+^ K^+^ ATPase—Chol concentration displays a regulatory effect—at low to moderate levels of cholesterol in the membrane (ratio of cholesterol: phospholipids from 1:4 to 1:2) there was determined an increase in activity, whereas at higher cholesterol concentrations (cholesterol: phospholipids ratio, 1:1) the enzyme activity decreases [28]. The function of rhodopsin also depends on the presence of cholesterol. High concentrations of cholesterol lead to a thickening of the layer and the inability of rhodopsin to change its conformation, absolutely necessary for its function [28,29].

The most important result is the demonstration of the condensing effect of cholesterol on hBest1 and composite hBest1 films. The condensing effect occurs at different cholesterol concentrations in both POPC/Chol/hBest1 and SM/Chol/hBest1 ternary monolayers. We show that Ca^2+^ ions play a key role for the cholesterol condensing effect—well pronounced for hBest1/Chol and hBest1/SM/Chol monolayers (Figure 1 and Figure 2). 

Among the common structural cholesterol-binding domains are CRAC (Cholesterol Recognition/interaction Amino acid Consensus sequence), CARC (“inverted” CRAC) and TP (tilted peptide) as one protein may have one, two or more cholesterol-binding sequences [44]. So far, there is no strong evidence to suggest that such domains exist in hBest1 structure. According to Lee et al., 2018, a possible interaction between cholesterol and hBest1 could exist, which would allow the spatial convergence of cholesterol with Asn70, Ser71, Ala73, Glu 74 [45]. In general, Chol could reveal its condensing effect bringing closer transmembrane domains of the protein or induce dimerization/oligomerization of protein molecules by direct interaction with its binding site/s [44]. Although, we observed a strong condensation effect in pure hBest1 monolayers only in the presence of Ca^2+^, it is not an evidence for direct binding of cholesterol to hBest1 (Figure 1A and Figure 2A).

The liquid ordered (L_o_) fractions of the cellular membrane are enriched with sphingolipids and cholesterol that exists in a significantly more condensed state in comparison with the liquid disordered (L_d_) fractions [46,47,48]. The effects of Ca^2+^ ions on the condensation process in hBest1/POPC/ Chol and hBest1/SM/ Chol composite monolayers indicate its role to the structural order of the films (Figure 1C,D and Figure 2B,C). This effect correlates well with our recent experiments made with hBest1 in live cells (where the levels of Ca^2+^ are notable), showing that hBest1 is preferentially localized not only in L_d_ state of the cell membranes, but its expression also increases the area of L_d_ fractions [41]. 

Because hBest1 prefers a less condensed phase in the membrane, it is possible that the membrane concentration of Chol affects protein activity (similar to Na^+^–K^+^ATPase, [28]), but most likely the biological functions of hBest1 depend on the overall change in physicochemical properties of the lipid environment, not just from the direct interactions with cholesterol molecules. 

## Supplementary Materials

The following are available online at https://www.mdpi.com/2077-0375/11/1/52/s1, Figure S1: BAM images of (**A**) POPC/Chol monolayers, (**B**) SM/Chol monolayers (at molar ratios of Chol 0.167; 0.285; 0.375; 0.444 and 0.5) in the presence or absence of Ca^2+^, at 35 ± 2 °C. The white scale bar = 100 µm.
