# Supplementary material for: Condensing Effect of Cholesterol on hBest1/POPC and hBest1/SM Langmuir Monolayers
Source: Membranes (Basel). 2021 Jan 13;11(1):52. doi: 10.3390/membranes11010052 (PMC7828479; doi:10.3390/membranes11010052)
Supplement: Supplementary file 1 [file membranes-11-00052-s001.pdf]

membranes

Supplementary Materials: Condensing Effect of Cholesterol on hBest1/POPC and hBest1/SM Langmuir Monolayers

Pavel Videv [1], Nikola Mladenov [1,2], Tonya Andreeva [3,4], Kirilka Mladenova [1], Veselina Moskova-Doumanova [1], Georgi Nikolaev [1], Svetla D. Petrova [1] and Jordan A. Doumanov [1,*]

1 Faculty of Biology, Sofia University "St. Kliment Ohridski", 8 Dragan Tzankov Blvd., 1164 Sofia, Bulgaria; pvidev@biofac.uni-sofia.bg (P.V.); nikola.mladenov@abv.bg (N.M.); k_mladenova@biofac.uni-sofia.bg (K.M.); moskova@biofac.uni-sofia.bg (V.M.-D.); gn_georgiev@uni-sofia.bg (G.N.); spetrova@biofac.uni-sofia.bg (S.D.P.)
2 Faculty of Medicine, Medical University-Sofia, 1 Sv. Georgi Sofiiski Str., 1431 Sofia, Bulgaria
3 Institute of Biophysics and Biomedical Engineering, Bulgarian Academy of Sciences, Acad. G. Bonchev Str., Bl. 21, 1113 Sofia, Bulgaria; t_andreeva@abv.bg
4 Faculty of Applied Chemistry, Reutlingen University, Alteburgstraße 150, 72762 Reutlingen, Germany
* Correspondence: doumanov@biofac.uni-sofia.bg; Tel.: +359 2 8167262

Citation: Videv, P.; Mladenov, N.; Andreeva, T.; Mladenova, K.; Moskova-Doumanova, V.; Nikolaev, G.; Petrova, S.D.; Doumanov, J.A. Condensing Effect of cholesterol on hBest1/POPC and hBest1/SM Langmuir monolayers. *Membranes* **2021**, *11*, 52. https://doi.org/10.3390/membranes11010052

Received: 26 December 2020
Accepted: 12 January 2021
Published: 13 January 2021

Publisher's Note: MDPI stays neutral with regard to jurisdictional claims in published maps and institutional affiliations.

Copyright: © 2021 by the authors. Licensee MDPI, Basel, Switzerland. This article is an open access article distributed under the terms and conditions of the Creative Commons Attribution (CC BY) license (http://creativecommons.org/licenses/by/4.0/).

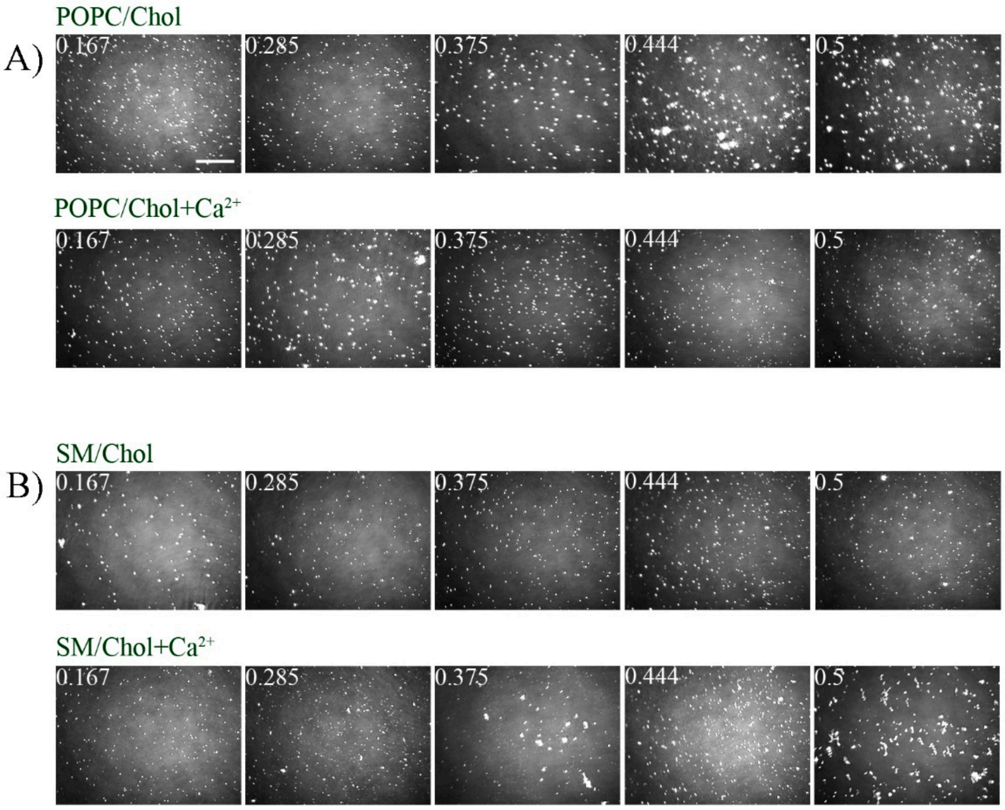

Figure S1. BAM images of (**A**) POPC/Chol monolayers, (**B**) SM/Chol monolayers (at molar ratios of Chol 0.167; 0.285; 0.375; 0.444 and 0.5) in the presentce or absence of Ca^{2+}, at 35 ± 2 °C. The white scale bar = 100 µm.
